# Supplementary material for: Enhanced detection of glioblastoma vasculature with superparamagnetic iron oxide nanoparticles and MRI
Source: Sci Rep. 2025 Apr 24;15:14283. doi: 10.1038/s41598-025-97943-y (PMC12022243; doi:10.1038/s41598-025-97943-y)
Supplement: Supplementary file 1 — Supplementary Material 1 [file 41598_2025_97943_MOESM1_ESM.docx]

**Supplementary Materials: Enhanced Detection of Glioblastoma Vasculature with Superparamagnetic Iron Oxide Nanoparticles and MRI**

Phillip W. Janowicz, Thomas Boele, Richard T. Maschmeyer, Yaser H. Gholami, Emma G. Kempe, Brett W. Stringer, Shihani P. Stoner, Marie Zhang, Taymin du Toit-Thompson^6^, Fern Williams^6^, Aude Touffu^6^, Lenka Munoz^4^, Zdenka Kuncic^2,8^, Caterina Brighi^1^ and David E. J. Waddington^1^


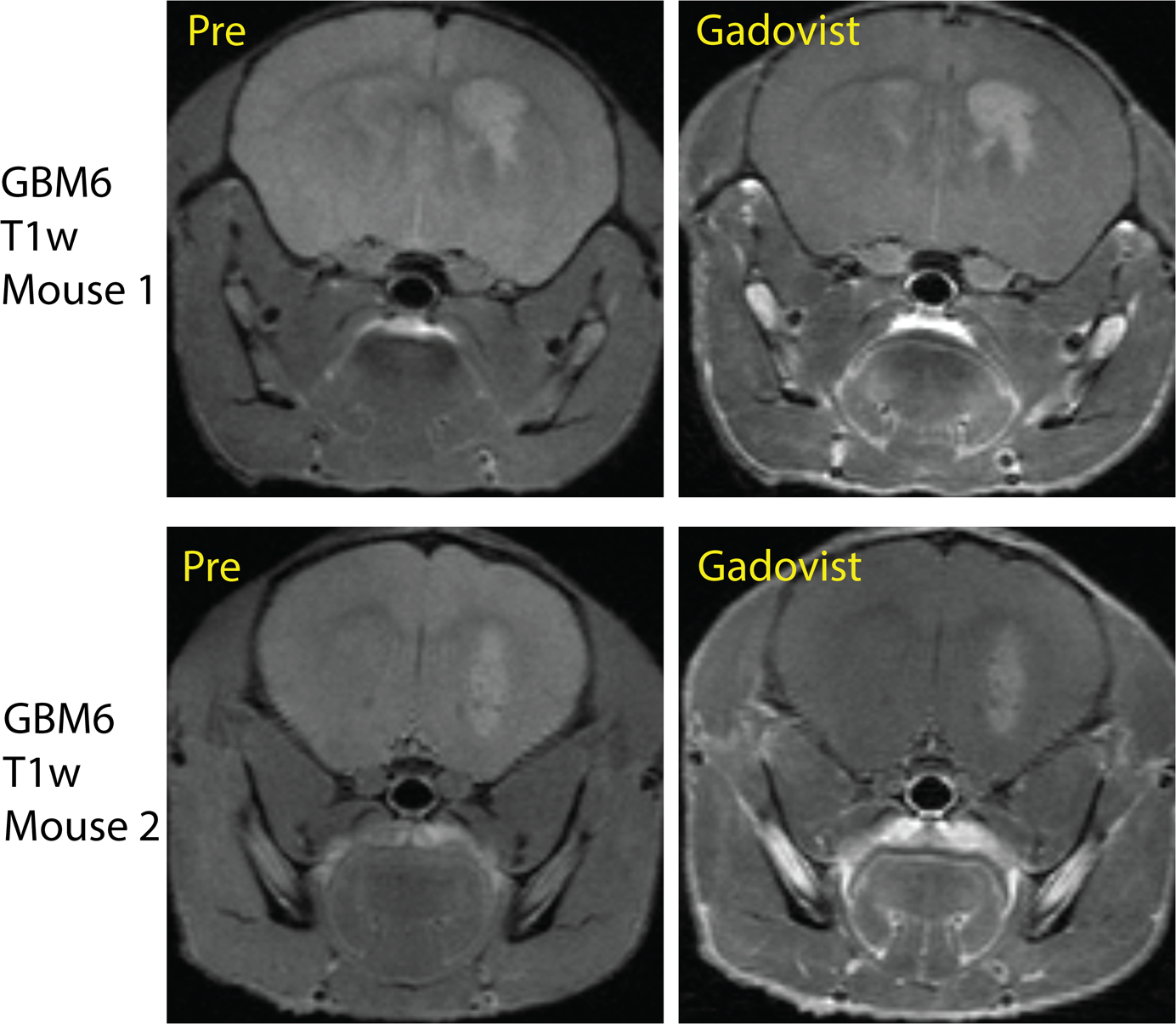


**Figure S1 –** Two examples of patient derived xenograft GBM6 pre- and post- Gadovist (0.1 mmol/kg), as shown in T1-weighted turbo spin echo scans. Permeability of the tumour BBB appears limited at this stage of growth due to modest Gadovist (Gadolinium) enhancement.


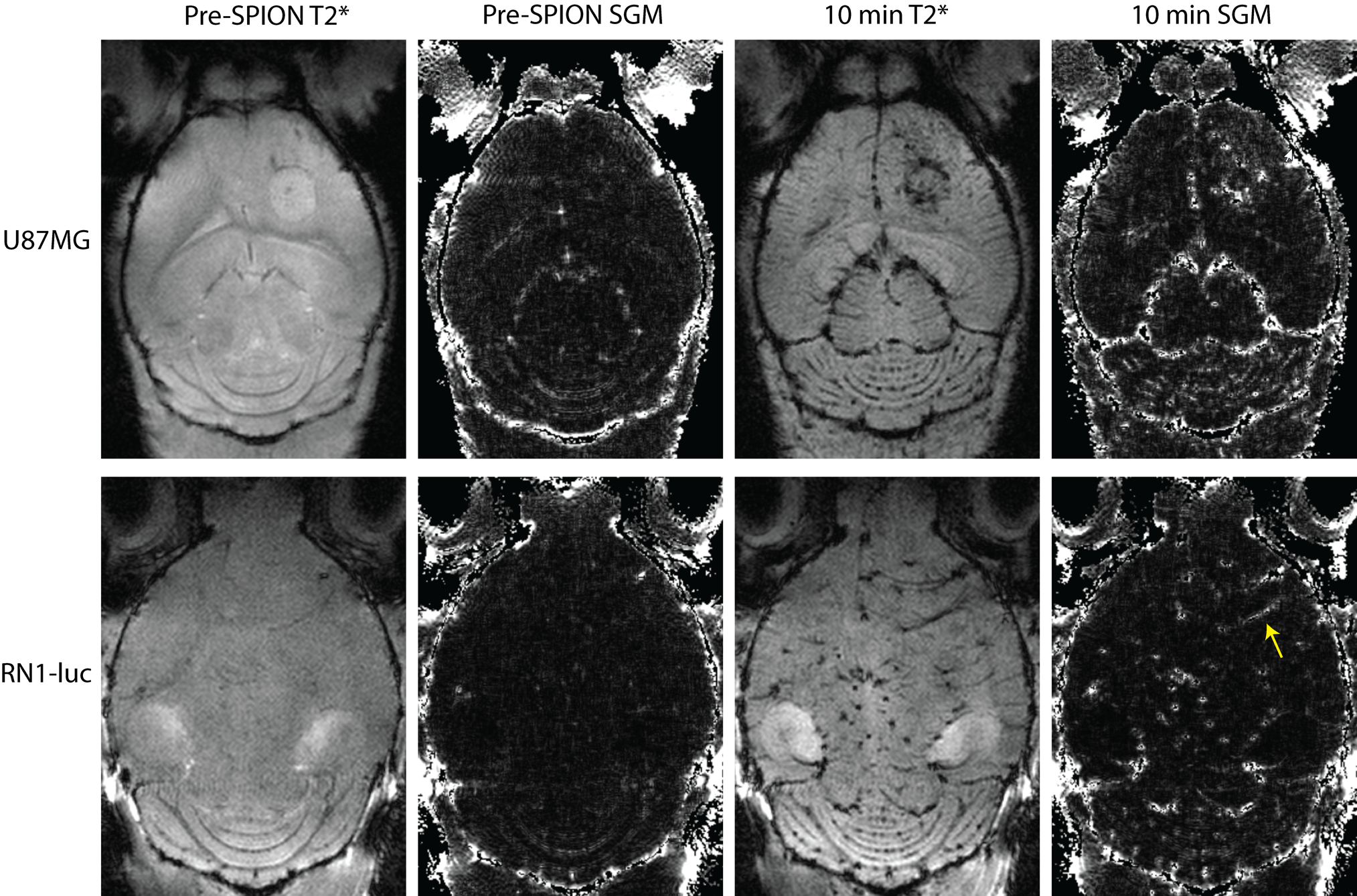


**Figure S2:** Additional susceptibility gradient mapping (SGM) images adjacent to source T2* gradient recalled echo sequences in U87MG and RN1-luc glioblastoma xenografts pre and post- 10 mg/kg PEG-SPION from a horizontal field of view. Yellow arrow points to an example of diffuse glioblastoma angiogenesis as shown by PEG-SPION positive contrast in SGM.


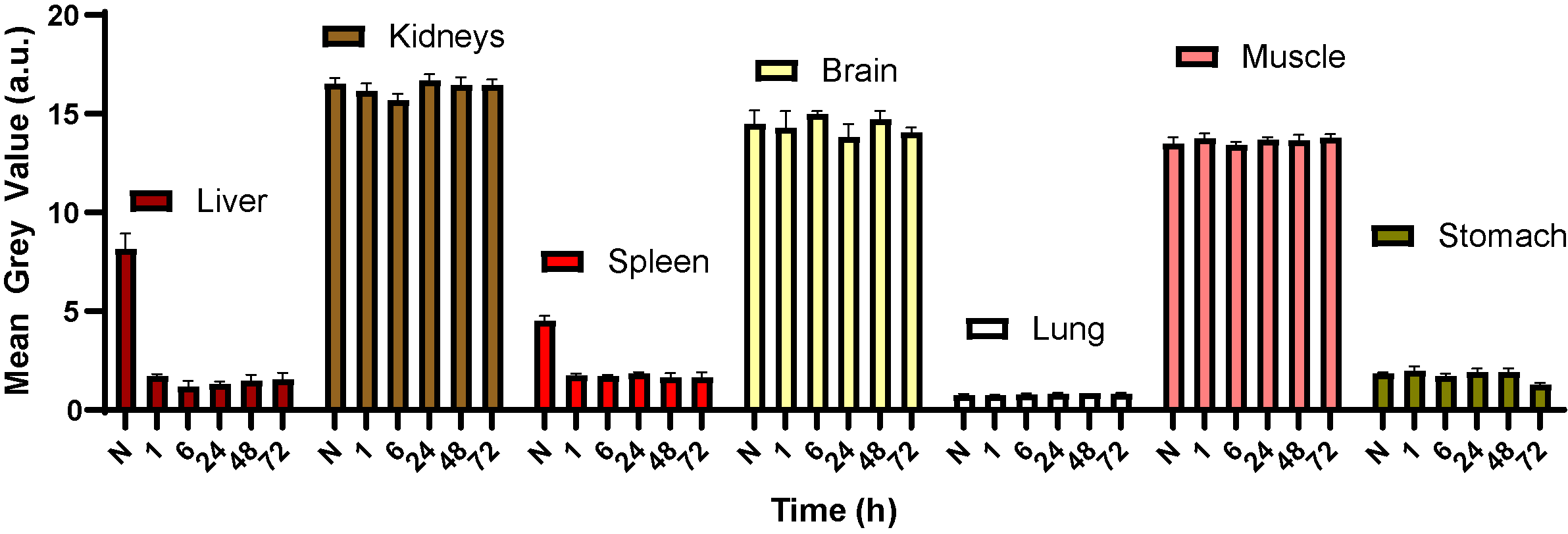


**Figure S3:** Mean + SEM pharmacokinetic biodistribution of 2 mg/kg ferumoxytol from T2*-weighted gradient recalled echo sequences (biological n=1, number of ROIs across slices per timepoint = 2 to 8).


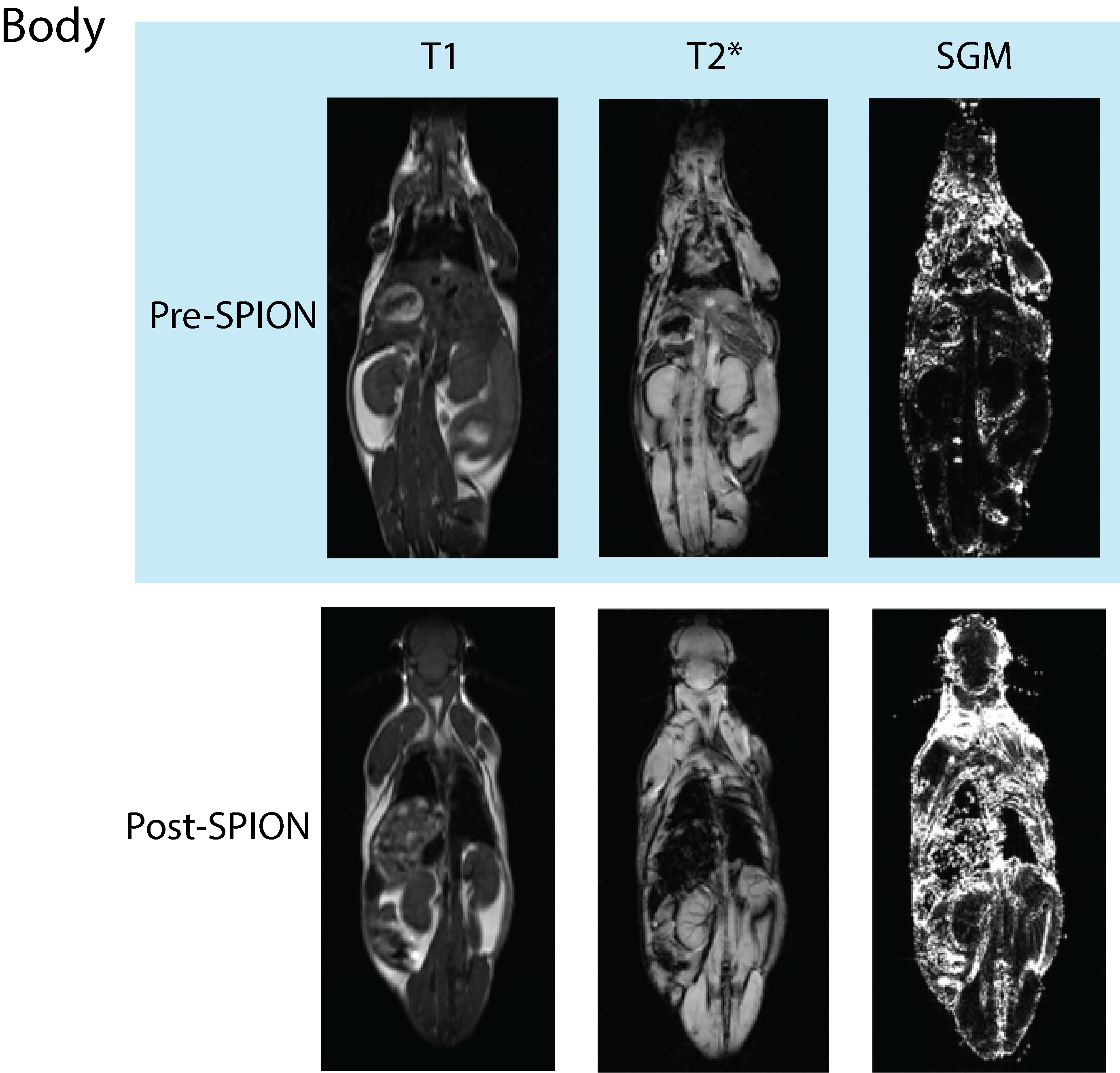


**Figure S4:** Mouse body 3 T T1 weighted and T2* weighted scans, plus susceptibility gradient maps pre and 60 minutes post 10 mg/kg PEG-SPION injection.


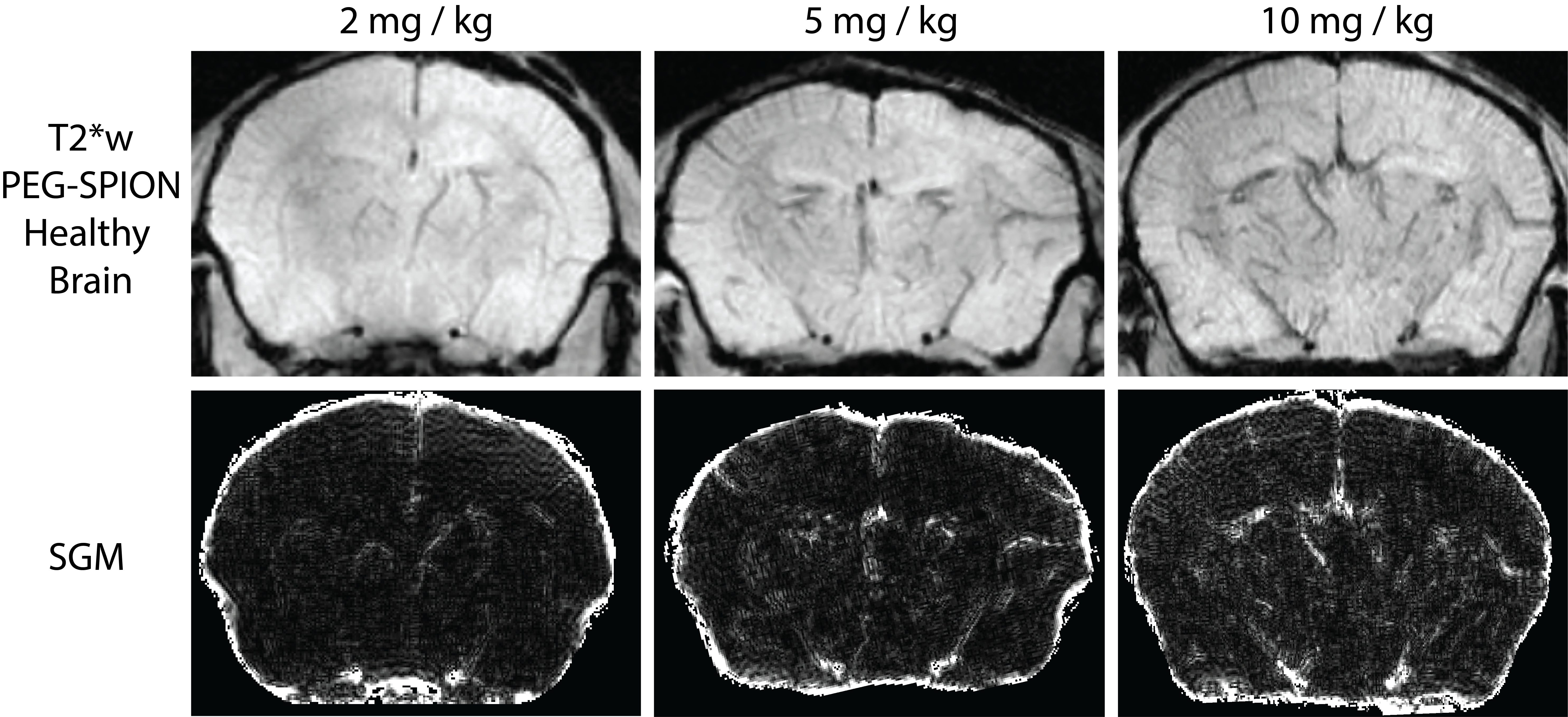


**Figure S5:** 7T T2* weighted images of healthy mouse brains 10 minutes post 2, 5 and 10 mg/kg PEG-SPION injection, showing a marked vascular T2* signal at all three doses. Susceptibility gradient maps (SGM) corresponding to the above first row images are shown in the second row.
